# Plasma Levels of Apelinergic System Components in Patients with Chronic and Acute Coronary Syndromes—A Pilot Study

**DOI:** 10.3390/jcm10194420

**Published:** 2021-09-26

**Authors:** Dorota Diakowska, Rafal Wyderka, Małgorzata Krzystek-Korpacka, Łukasz Osuch, Anna Leśków, Alicja Sołtowska, Marta Stanek, Joanna Rosińczuk, Joanna Jaroch

**Affiliations:** 1 Department of Clinical Nursing, Faculty of Health Science, Wroclaw Medical University, Bartla 5, 51-618 Wroclaw, Poland; anna.leskow@umed.wroc.pl (A.L.); joanna.rosinczuk@umed.wroc.pl (J.R.); 2 Department of Cardiology, Tadeusz Marciniak Lower Silesia Specialist Hospital—Emergency Medicine Center, Fieldorf 2, 54-049 Wroclaw, Poland; ramwyder@gmail.com (R.W.); losuch93@gmail.com (Ł.O.); ala.soltowska@gmail.com (A.S.); joanna.jaroch@umed.wroc.pl (J.J.); 3 Department of Medical Biochemistry, Wroclaw Medical University, Chalubinskiego 10, 50-368 Wroclaw, Poland; malgorzata.krzystek-korpacka@umed.wroc.pl; 4 Department of Internal Nursing, Faculty of Health Science, Wroclaw Medical University, Bartla 5, 51-618 Wroclaw, Poland; 5 Lower Silesian Center of Blood Donation and Therapy, Red Cross 5/9, 50-345 Wroclaw, Poland; marta.stanek@rckik.wroclaw.pl

**Keywords:** coronary artery disease, chronic coronary syndrome, acute coronary syndrome, apelinergic system

## Abstract

The effects of the apelinergic system components apelin (AP) and elabela (ELA) in the regulation of human cardiovascular homeostasis, and data concerning the relationship between ELA and AP and coronary artery disease (CAD) are yet unknown. The aim of the study was the evaluation of AP, ELA and APJ-receptor levels in the plasma of patients with chronic coronary syndromes (CCS) and acute coronary syndromes (ACS). The study group consisted of 114 patients with CAD and 33 healthy controls. Patients were divided into two groups: with CCS (*n* = 30) and ACS (*n* = 84). Routine laboratory tests and plasma ELA, AP-17, AP-13 and APJ receptor levels were measured. Echocardiographic data were analyzed in all patients. Levels of AP-17 and ELA were significantly lower in CCS than in healthy controls and ACS patients. We demonstrated significant increase of levels of plasma apelinergic system peptides, especially ELA and AP-17 in ACS patients compared with healthy controls and CCS, suggestive of compensating up-regulation mechanisms. There is a relationship between circulating ELA and AP-17 levels and classical, biochemical markers of ischemia and left ventricular ejection faction as well.

## 1. Introduction

The main cause of coronary artery disease (CAD) is atherosclerosis. This is a multifactorial process resulting from an excessive inflammatory response to various injurious stimuli to the arterial wall [1,2].

Adipokines, bioactive products of subcutaneous or visceral adipose tissue, were shown to play an important role in modulation and pathogenesis of the cardiovascular system [3]. They are secreted into the bloodstream, from where they enter the heart muscle and the walls of large arteries [3]. Among those, components of the newly-discovered apelinergic system may participate in the development of atherosclerosis, hypertension, myocardial infarction and heart failure [4,5,6,7].

The apelinergic system consists of two peptide ligands: apelin (AP) and Elabela (ELA) and a single class A G-protein coupled apelin receptor (APJ receptor). Each ligand may be processed into a variety of isoforms endogenously, with AP ranging from 13 to 55 amino acids and ELA from 11 to 32 [5,8,9]. AP and ELA are peptides which can be produced by cells of adipose tissue, lung, gastrointestinal tract and central nervous system tissues, cardiomyocytes, non-cardiomyocytes, fraction of fibroblasts and the endothelium of large arteries and coronary vessels [10,11,12]. It has been proven that ELA participates in early cardiac development during zebrafish embryogenesis by inducing cardiogenesis and vasculogenesis [13,14].

In laboratory studies, AP and ELA were found to reduce blood pressure, increase cardiac contractility, stimulate angiogenic and vasodilatatory effects, and influence anti-atherosclerotic and anti-oxidative components. [4,8,10,11,15,16,17]. AP and ELA have some comparable functions, but physiological effects of ELA are stronger [4,8]. However, the effects of AP and ELA in the regulation of human cardiovascular homeostasis, and data concerning the relationship between ELA and AP and CAD, remain unknown.

The aim of the study was the comprehensive evaluation of apelinergic system components in the plasma of patients with chronic or acute coronary syndromes. The relationship between ELA, AP-13, AP-17, APJ receptor and selected markers of myocardial infarction was investigated. Finally, we also aimed to verify whether circulating components of the apelinergic system might become new biomarkers of myocardial infarction.

## 2. Material and Methods

### 2.1. Patients Characteristic

A group of 114 patients with diagnosed coronary artery disease (CAD), who were admitted to the Tadeusz Marciniak Lower Silesia Specialist Hospital—Emergency Medicine Center, Department of Cardiology (Wroclaw, Poland), was enrolled into the study. There were 90 (79%) men and 24 (21%) women with mean age 60.1 ± 8.6 years old. Patients with CAD were divided into two groups: with chronic coronary syndrome (CCS) (*n* = 30) and acute coronary syndrome (ACS) (*n* = 84).

As a reference we used sera from 33 blood donors, obtained from Lower Silesian Center of Blood Donation and Therapy, Wroclaw, Poland. All blood donors were considered healthy on the basis of physical examination and routine blood tests. The control group consisted of 29 (88%) men and 4 (12%) women with a mean age 57.9 ± 4.4 years old. Demographical characteristics of the study and control groups are presented in Table 1. There were insignificant differences in gender distribution (*p* = 0.336), mean age (*p* = 0.278) and BMI factor (*p* = 0.739) between study and control groups.

Patients with chronic coronary syndrome were referred to coronary angiography due to stable angina pectoris symptoms (CCS class II or III). Exclusion criteria for the CCS group were as follows: a history of heart failure or LVEF < 50%, severe valvular heart disease or cardiomyopathies (hypertrophic, dilated) diagnosed by echocardiography, uncontrolled hypertension (blood pressure values > 180/110 mmHg; after lowering of blood pressure patient could enter the study), and implantation of pacemaker. Medical treatment of stable coronary disease was applied according to the Guidelines of the European Society of Cardiology (ESC).

Patients with no history of myocardial infarction, diagnosed with acute coronary syndrome following the criteria of the 4th universal definition according to the recent Guidelines of the European Society of Cardiology (2018) were classified into the acute coronary syndrome group (ACS). In the group of ACS patients, only subjects with MI and not with unstable angina entered the study.

### 2.2. Ethical Consideration

The study protocol was approved by the Bioethics Committee of Wroclaw Medical University, Poland (signature number KB-749/2020). Informed consent was obtained from all subjects.

### 2.3. Biochemical Determinations

Venous blood samples (2 mL) were collected after overnight fasting, at least 24 h after admission, for routine laboratory tests by vacuum system. In case of patients with acute coronary syndrome, the blood sample was collected on the first day of hospitalization. The blood samples were collected into tubes containing EDTA clotted for 30 min. at room temperature, then were centrifuged at 3000× *g* (centrifuge MPW 260R, MPW Med. Instruments, Warszawa, Poland) for 15 min at room temperature. Obtained sera were stored at −20 °C.

Concentrations of ELA, AP-13, AP-17 and APJ receptors were measured by ELISA kits (all tests were from MyBioSource Inc., San Diego, CA, USA), according to the manufacturer’s instructions. The sensitivity of the ELA assay was 10.0 pg/mL. The sensitivity of the APJ receptor was 7.4 pg/mL, while intra- and inter-assay CVs were <8.0% and <10.0%, respectively. The sensitivity of the AP-13 assay was less than 33.0 pg/mL, and intra- and inter-assay CVs were <4.0% and <6.5% respectively. The minimum detectable dose of AP-17 was less than 72.0 pg/mL, intra- and inter-assay CVs: <10.0% and <12.0% respectively.

Data on the hematological variables, glucose, total cholesterol (TCh), HDL and LDL cholesterol, triglicerides (TG), C-reactive protein (CRP), high sensitive troponin T (Hs-Troponin T), creatinine kinase myocardial band (CK-MB) and N-terminal pro-B-type natriuretic peptide (NT-proBNP) was retrieved from patients’ medical records.

### 2.4. Echocardiographic Data

In all patients the following echocardiographic parameters were obtained using cardiovascular ultrasound system VIVID 9-GE (GE Healthcare, Chicago, IL, USA): left ventricular hypertrophy and remodeling parameters; left ventricular end-diastolic volume; interventricular septum thickness; posterior wall thickness; left ventricular mass; left ventricular systolic function indices: ejection fraction (Simpson method); wall motion score index; stress/strain parameters; eccentricity index and left ventricular diastolic function indices from mitral inflow and tissue doppler imaging (TDI); left atrial volume.

### 2.5. Statistical Analysis

All data were statistically analyzed using Statistica v. 13.3 software (Tibco Software Inc., Palo Alto, CA, USA). Categorical variables were presented as a number of observation and percentage, continuous variables as mean ± standard deviation (±SD). Distribution of data was tested with the Shapiro-Wilk normality test. The power of the sample size to detect the association at *p* < 0.05 was calculated. Independent samples were analyzed using the Student’s *t* test and one-way ANOVA analysis with post-hoc Tukey test. Qualitative data were analyzed using Pearson’s chi-square test or Fisher’s exact test. Pearson correlation coefficients (ρ) were calculated to evaluate associations between pairs of variables.

Receiver operating characteristic (ROC) analysis with calculation of area under the ROC curve (AUC) was performed for analysis of diagnostic potential of apelinergic system components. The accuracy, sensitivity, specificity, positive predictive value (PPV), negative predictive value (NPV), likelihood ratio of positive results (LR (+)), likelihood ratio of negative results (LR (−)) and Youden’s index were also obtained.

In all analyzes 2-tailed *p*-value of *p* < 0.05 was considered statistically significant.

## 3. Results

### 3.1. Basic Characteristic of Patients with CCS and ACS

The clinical, laboratory and echocardiographic characteristics of patients with CCS and ACS are presented in Table 2. The concentrations of myocardial infarction (MI) markers such as hs-Troponin T, CK-MB and NT-proBNP were significantly higher in the ACS group than in the CCS group (*p* < 0.001 for all). The level of LVEF significantly decreased in ACS compared with the CCS group (*p* < 0.0001). The concentrations of TCh, LDL and CRP were significantly higher in ACS subjects than in CCS (*p* < 0.0001 for all). The tendency to significantly higher HDL concentration was showed in patients with CCS (*p* = 0.051). In ACS groups we observed significantly higher rates of smoking history (*p* < 0.001). There were no significant differences in levels of Hg, Glu, TG and family history of CAD between the two groups.

### 3.2. Circulating Levels of ELA, AP-13, AP-17 and APJ Receptor in CAD Patients and Healthy Controls

We compared the concentrations of plasma ELA, AP-13, AP-17 and APJ receptors in sera in CCS, ACS, and the control groups. As presented in Table 3, statistically significant differences in levels of all apelinergic system components were found between CCS, ACS and controls subjects (*p* < 0.0001 or *p* = 0.004). The concentrations of plasma ELA and AP-17 were significantly higher in the patients with ACS than in CCS and control group (*p* < 0.05 for all). On the contrary, levels of ELA and AP-17 were significantly lower in the CCS group compared with the healthy control (*p* < 0.05). Level of circulating APJ receptors was significantly lower in patients with ACS than in CCS, but it was significantly higher than in the control group (*p* < 0.05). The concentration of plasma AP-13 was significantly higher in ACS subjects than in the healthy control (*p* < 0.05), but no significant difference in concentration of plasma AP-13 between CCS and the ACS group was observed.

We analyzed associations between components of the apelinergic system and biochemical markers of myocardial infarction (MI) in all patients (Table 4). We observed statistically significant positive correlations between the concentrations of plasma ELA or AP-17 levels and levels of hs-troponin T and CK-MB (*p* < 0.05 for all). Significantly negative correlations were found between plasma ELA or AP-17 and LVEF values (*p* < 0.0001 for both). There was no significant association between AP-13 or APJ receptor levels and biochemical markers of MI.

### 3.3. Diagnostic Potential of Circulating ELA and AP-17 Levels in Acute Coronary Syndrome in CAD Patients

**Figure 1:**
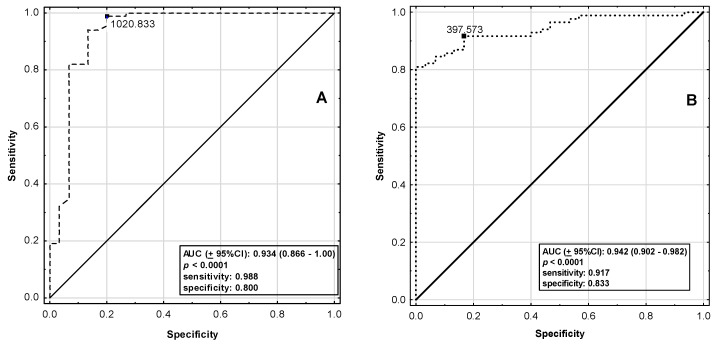
ROC curve for (**A**) ELA and (**B**) AP– 17 as a biomarkers of myocardial infarction (dashed line).

To assess the strength of correlation and probable diagnostic properties of ELA and AP-17 as markers of myocardial infarction, an ROC analysis was performed (Table 5). A circulating ELA concentration at a cut-off point above 1020.83 pg/mL may be a good prognostic marker of acute coronary syndrome (AUC = 0.934, sensitivity = 0.988, specificity = 0.800) (Table 5 and Figure 1A). The circulating AP-17 at a cut-off point above 397.57 pg/mL (AUC = 0.942, sensitivity = 0.917, specificity = 0.833) (Table 5 and Figure 1B) also appears to be a beneficial prognostic marker of ACS.

## 4. Discussion

The fundamental mechanism of CAD development is extremely complex, and involves a combination of endothelial dysfunction, inflammation, extensive lipid deposition in the intima, proliferation of vascular smooth muscle cells and the activation of platelets [1].

This is the first study evaluating levels of the apelinergic system components in patients with diagnosed CAD—both chronic and acute coronary syndromes.

### 4.1. Levels of Components of the Apelinergis System in Chronic Coronary Syndromes

We observed that levels of AP17 and ELA were significantly lower in patients with CCS than in ACS and the control groups. On the contrary, concentration of the APJ receptor was significantly higher in the CCS group than in ACS and the controls.

Our results are consistent with previous studies that found lower apelin levels in patients with stable coronary heart disease compared with controls [18,19]. Apelin was shown to attenuate the progression of atherosclerosis by promoting cholesterol efflux and reducing foam cell formation [20]. Other studies showed an atheroprotective effect of apelin. In an Apo E-deficient atherosclerosis model apelin treatment mitigated native and Ang II-accelerated atherosclerosis by promoting nitric oxide (NO) production [21]. There is some evidence demonstrating that ELA and AP have an antihypertensive effect [4,8,22,23,24,25].

Similar to our results, circulating ELA levels were reduced in patients with essential hypertension in the study by Li et al. [22]. They reported that decrease of plasma ELA levels was associated with endothelial dysfunction and a reduction of ELA synthesis and release from hypertension-related endothelium [22]. In patients with chronic coronary syndrome, the protective role of the AP/ELA/APJ receptor axis may be decreased due to endothelial cell injury during increase of blood pressure and inhibition of AP and ELA expression [22].

### 4.2. Levels of Components of the Apelinergic System in Acute Coronary Syndromes

We demonstrated significant increase of levels of plasma apelinergic system peptides, especially ELA and AP-17, in ACS subjects compared with the healthy control and CCS groups. Our findings are in accordance with previous studies, where significantly higher ELA levels were observed in ACS patients, STEMI patients or in patients with complete heart blockage [26,27,28]. In the study by Du et al. [27] the authors found that plasma levels of ELA were higher in patients with ACS than in controls without coronary lesions. There was no significant correlation between plasma ELA level and the number of diseased coronary arteries; however, a nonlinear relationship between ELA and Syntax scale was detected [27]. In animal models, ELA had positive inotropic function and protected against MI. Animal studies revealed that the ELA level was upregulated in post-infarction cardiac remodeling and was correlated with LVEF [29]. Treatment of FC-EL1-21 fusion mitigated cardiac dysfunction in MI rats by increasing angiogenesis and promoting cardiomyocyte proliferation [30].

ELA levels may reflect the vulnerability of atheromatic plaques in ACS. These results suggest a potential role for peptides of the apelinergic system in pathogenic processes of acute coronary syndrome. We suggest that high ELA, AP-17, APJ receptor levels in the patients with MI might have a protective effect against CAD progression and vascular damage.

Apelin is a protective factor against ACS [31,32]. Earlier studies showed that patients with STEMI with decreased apelin concentration in plasma had a higher incidence of adverse cardiac events after percutaneous coronary intervention [32]. Our study demonstrated significant increase of plasma AP-17 and AP-13 in ACS patients in comparison with the control group.

Correlation analyzes between each of apelinergic system components and biochemical diagnostic and prognostic markers of myocardial infarction (CRP, Hs-Troponin T, CK-MB, NT-proBNP) as well as echocardiographic (LVEF) were performed in order to clarify whether the ELA/AP/APJ receptor axis is implicated in vascular and myocardial dysfunction. We showed significant positive association between circulating ELA and AP-17 levels and plasma Hs-Troponin T, CK-MB. Significant negative correlation was observed between ELA or AP-17 and LVEF values. The same results were showed in the Donmez et al. study [26], where significant positive correlation between ELA and the creatinine kinase myocardial band (CK-MB) or N-terminal pro-B-type natriuretic peptide (NT-proBNP) was demonstrated. Toczylowski et al. reported the increase of mRNA expression of apelin in subcutaneous adipose tissue of patients with impaired left ventricular diastolic function [33]. Rakhshan et al. have shown that cardiac function measured by echocardiography significantly improves after ELA infusion in acute myocardial infarction in rats [17]. Therefore, they suggest that ELA may have a therapeutic potential in acute cardiovascular diseases.

ROC analysis demonstrated that ELA and AP-17 showed potential as markers of ACS.

There is a need for better understanding of the interactions between the components of the apelinergic system and their impact on vascular and cardiac dysfunction in chronic coronary syndromes and conversion to acute coronary syndromes as well.

### 4.3. Limitations of the Study

This was a cross-sectional, single-center, male-biased study. The study group consisted only of the Caucasian population, and the ELA and AP-17 levels were tested only once, on the first day of hospitalization. It is necessary to conduct a larger study with sequential assays to evaluate the pathophysiology of the apelinergic system.

## 5. Conclusions

Levels of AP-17 and ELA were significantly lower in chronic coronary syndromes than in healthy controls and acute coronary syndrome patients. We demonstrated significant increase of levels of plasma apelinergic system peptides, especially ELA and AP-17 in acute coronary syndrome patients compared with healthy controls and chronic coronary syndromes, suggestive of compensating up-regulation mechanisms. There is a relationship between circulating ELA and AP-17 levels and classical, biochemical markers of ischemia and left ventricular ejection faction as well.

## Figures and Tables

**Table 1:** Demographical characteristic of CAD patients in both groups and healthy controls. Descriptive data are presented as number (percentage) or mean (+SD).

Variable	Healthy Control (*n* = 33)	Chronic Coronary Syndrome (CCS) (*n* = 30)	Acute Coronary Syndrome (ACS) (*n* = 84)	*p*-Value (Fischer Exact Test or ANOVA)
Gender: Male Female	29 (87.9) 4 (12.1)	22 (73.3) 8 (26.7)	68 (80.9) 16 (19.1)	0.336
Age (Years)	57.91 ± 4.40	61.13 ± 8.42	59.11 ± 8.96	0.278
BMI (kg/m^2^)	24.83 ± 2.38	27.11 ± 2.72	27.17 ± 3.96	0.739

**Table 2:** Clinical, biochemical and echocardiographic characteristic of patients with chronic and acute coronary syndrome. Descriptive data are presented as number (percentage) or mean (±SD).

Variable	Chronic Coronary Syndrome (CCS) (*n* = 30)	Acute Coronary Syndrome (ACS) (*n* = 84)	*p*-Value (Chi-Square, Fischer Exact or Student *t* Tests)
Smoking History:			0.001 *
No	24 (80.0)	37 (44.0)	
Yes	6 (20.0)	47 (56.0)	
Family History of CAD:			0.633
No	20 (66.7)	60 (71.4)	
Yes	10 (33.3)	24 (28.6)	
Hg (g/dL)	13.91 ± 1.38	14.32 ± 2.19	0.345
Glu (mg/mL)	122.82 ± 39.69	136.68 ± 53.84	0.214
TCh (mg/mL)	146.80 ± 39.76	180.84 ± 36.29	<0.0001 *
HDL (mg/mL)	49.00 ± 11.25	44.27 ± 10.82	0.051 ^#^
LDL (mg/mL)	71.72 ± 35.55	109.16 ± 39.99	<0.0001 *
TG (mg/mL)	120.26 ± 54.53	146.52 ± 80.06	0.105
CRP (mg/L)	2.91 ± 1.28	13.72 ± 9.12	<0.0001 *
Hs-Troponin T (ng/L)	17.25 ± 16.41	3081.27 ± 2387.14	<0.0001 *
CK-MB (IU/L)	15.69 ± 6.51	115.82 ± 102.76	<0.0001 *
NT-proBNP (pg/mL)	169.57 ± 110.05	835.76 ± 646.38	0.007 *
LVEF (%)	59.63 ± 4.68	44.72 ± 7.72	<0.0001 *
STEMI:			-
No	-	30 (35.7)	
Yes	-	54 (64.3)	

*: statistically significant; ^#^: tendency to statistically significant value.

**Table 3:** Concentration of plasma ELA, AP-13, AP-17 and APJ receptor levels in CCS and ACS groups and in healthy controls. Descriptive data are presented as mean (±SD).

Variable	Healthy Control (*n* = 33) (A)	Chronic CAD (CCS) (*n* = 30) (B)	Acute Coronary Syndrome (ACS), (*n* = 84) (C)	*p*-Value (ANOVA)
Elabela (pg/mL)	1176.00 ± 237.52 ^B,C^	784.30 ± 371.70 ^A,C^	1477.82 ± 229.83 ^A,B^	<0.0001 *
Apelin-13 (pg/mL)	59.61 ± 14.35 ^C^	62.67 ± 10.46	69.18 ± 15.97 ^A^	0.004 *
Apelin-17 (pg/mL)	511.09 ± 85.74 ^B,C^	341.68 ± 90.44 ^A,C^	627.34 ± 168.66 ^A,B^	<0.0001 *
APJ Receptor (pg/mL)	1018.77 ± 333.41 ^B,C^	1510.90 ± 205.90 ^A,C^	1383.28 ± 204.97 ^A,B^	<0.0001 *

*: statistically significant; letters ^A, B, C^ indicate statistically significant differences (*p* < 0.05) between two groups estimated by post-hoc Tukey’s test.

**Table 4:** Correlations between apelinergic system components and markers of myocardial infarction in total group of CAD patients.

Variables	Correlation	ELA (pg/mL)	AP-13 (pg/mL)	AP-17 (pg/mL)	APJ Receptor (pg/mL)
Hs-Troponin T (ng/mL)	r *p*-Value	0.336 0.001 *	−0.042 0.691	0.293 0.005 *	−0.069 0.520
CK-MB (IU/L)	r *p*-Value	0.387 <0.0001 *	0.043 0.685	0.407 <0.0001 *	0.050 0.640
NT-proBNP (pg/mL)	r *p*-Value	0.143 0.180	0.023 0.826	0.194 0.069	−0.151 0.157
LVEF (%)	r *p*-Value	−0.537 <0.0001 *	−0.097 0.363	−0.417 <0.0001 *	0.059 0.582

*: statistically significant; r: correlation coefficient

**Table 5:** Diagnostic potential of ELA and AP-17 as indicators of myocardial infarction.

	ELA	AP-17
AUC (±95%CI)	0.934 (0.866–1.00)	0.942 (0.902–0.982)
*p*-Value	<0.0001	<0.0001
SE	0.035	0.020
Cut-Off Point (pg/mL)	1020.83	397.57
Sensitivity	0.988	0.917
Specificity	0.800	0.833
Accuracy	0.939	0.895
LR (+)	0.200	0.167
LR (−)	0.012	0.083
PPV	0.933	0.939
NPV	0.960	0.781
Youden’s Index	0.788	0.750

AUC: area under ROC curve, 95%CI: 95% confidence interval, SE: standard error, LR (+): likelihood ratio of positive results, LR (−): likelihood ratio of negative results, PPV: positive predictive value, NPV: negative predictive value.

## Data Availability

The data are available from corresponding author and may be shared if necessary.
